# Supplementary material for: What Are the Key Workplace Influences on Pathways of Work Ability? A Six-Year Follow Up
Source: Int J Environ Res Public Health. 2019 Jul 3;16(13):2363. doi: 10.3390/ijerph16132363 (PMC6651683; doi:10.3390/ijerph16132363)
Supplement: Supplementary file 1 [file ijerph-16-02363-s001.pdf]

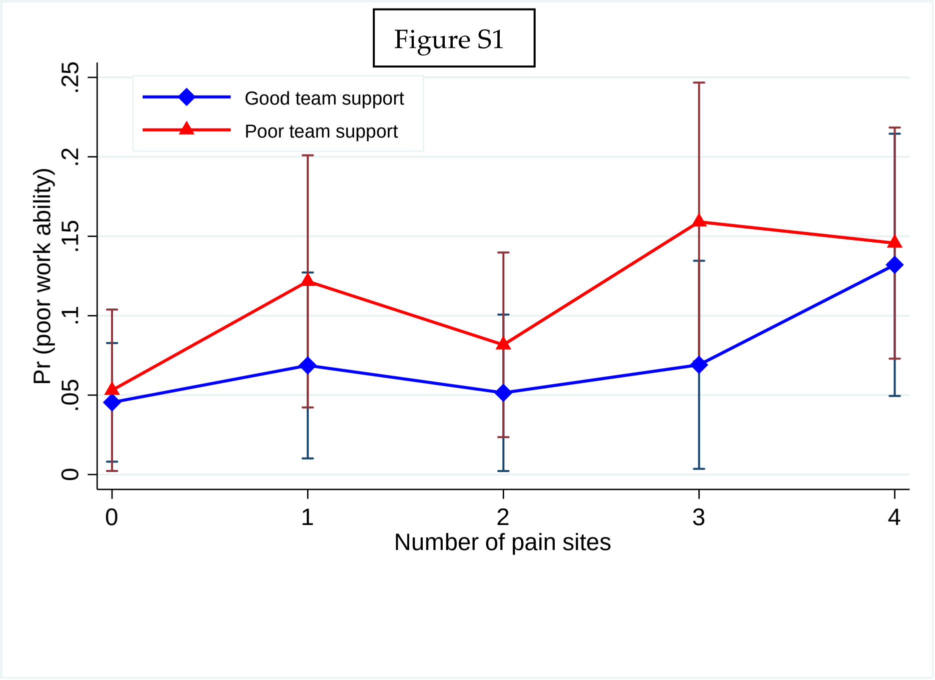

Figure S1: Predictive probability of trajectory of poor work ability due to number of pain sites and team support. Predictive margins with their 95% CIs.

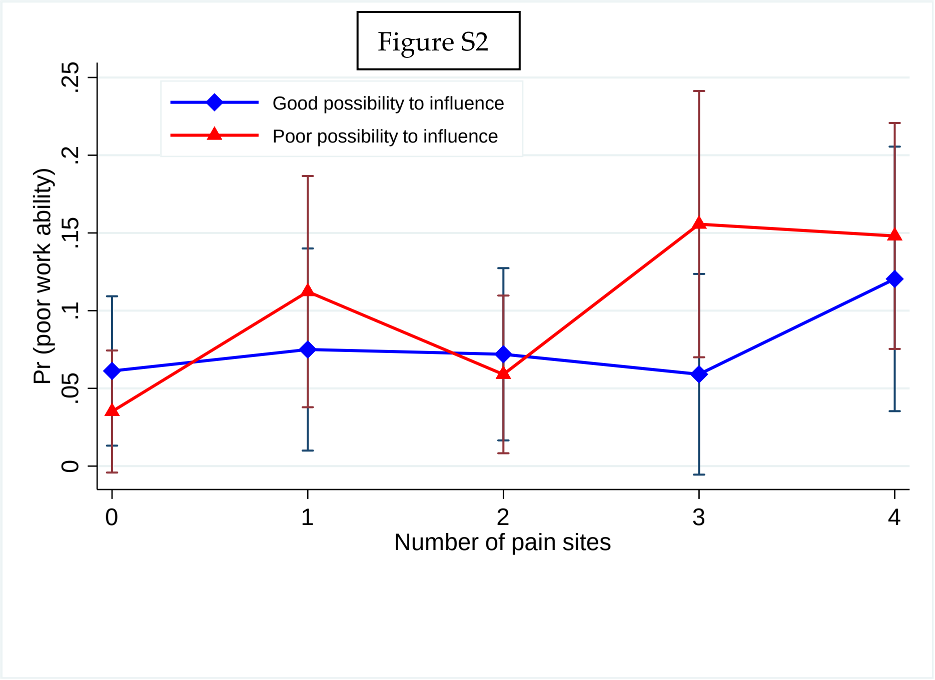

Figure S2: Probability of poor work ability pathway due to number of pain sites and possibility to influence at work. Predictive margins with 95% CIs.

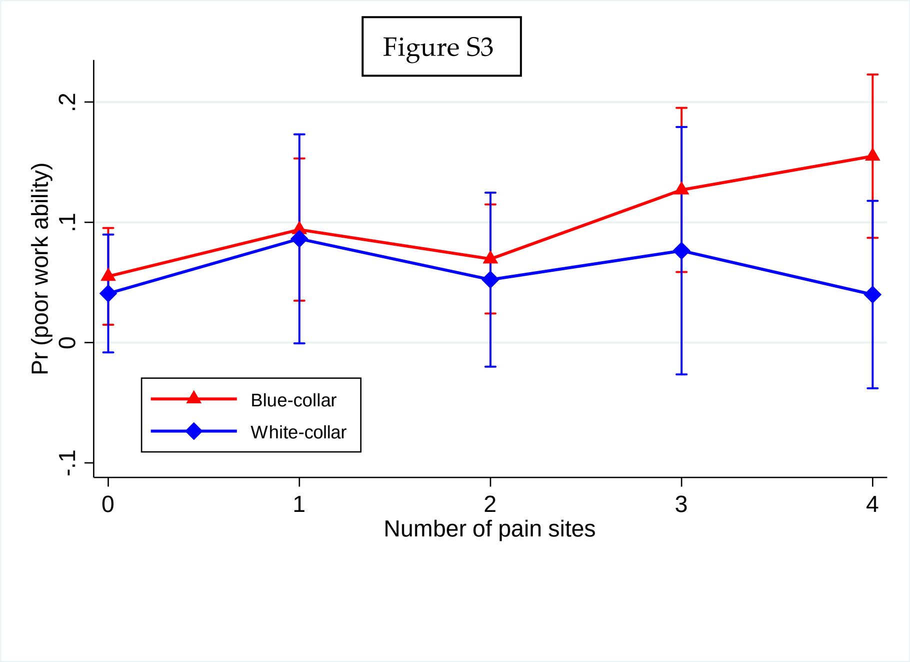

Figure S3: Probability of a poor work ability pathway due to number of pain sites and occupational class. Predictive margins with 95% CIs.
